# BCG and beyond: unlocking new frontiers in TB vaccine development

**DOI:** 10.3389/fimmu.2025.1608104

**Published:** 2025-07-30

**Authors:** Aishwarya Shaji, Akanksha Verma, Ashima Bhaskar, Ved Prakash Dwivedi

**Affiliations:** Immunobiology Group, International Centre for Genetic Engineering and Biotechnology, New Delhi, India

**Keywords:** tuberculosis, BCG, trained immunity, vaccine, host genetics

## Abstract

With over 10 million new cases and 1.6 million deaths annually, tuberculosis (TB) continues to be a significant worldwide health-burden. To assist in curbing the spread of TB, the century-old BCG, which is a live-attenuated vaccine, is now the only licensed TB vaccine used in humans. However, BCG’s limited efficacy and poor antigenicity in adults have evoked the need to design new vaccines against TB. The limited parameter is the availability of potent antigens; as a consequence, it is imperative to study the *Mycobacterium tuberculosis* (*Mtb*)-specific antigens that can provide a stronger immune response if included in vaccine candidates. Through this review, we aim to concentrate on the progress of current vaccine-candidates undergoing preclinical and clinical-studies. Moreover, it is not the pathogen but the genetics of the host that plays an essential role in fine tuning the immune-response and susceptibility to TB. Over the past 50 years, a systematic approach to treating TB patients has overlooked factors like pharmacokinetics, immune-response, and treatment duration. Henceforth, this review highlights the precision medicine-guided approach considering genetic makeup and host immunity that could influence clinical management choices. The consolidated review will shed light on advancements in vaccine-candidates, which can be harnessed in prophylactic development against TB.

## Graphical Abstract

BCG vaccine efficacy, immunogenicity, and limitations necessitate better TB vaccine candidates that account for host genetics and precision medicine.

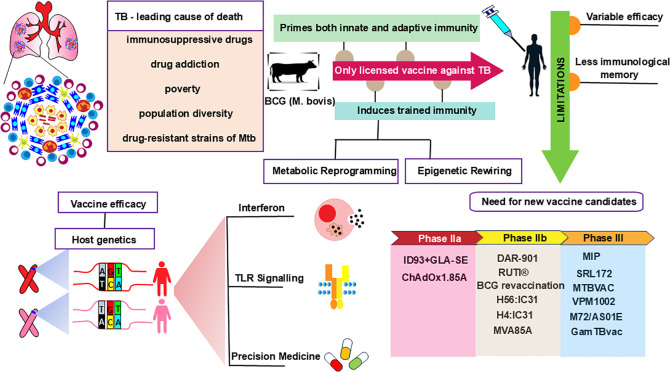

## 1 Introduction

Tuberculosis (TB) was the leading cause of death due to a single infectious agent worldwide before the Coronavirus disease 2019 (COVID-19) pandemic, which was brought on by the severe acute respiratory syndrome coronavirus 2 (SARS-CoV-2) (1). A new era in TB prevention, diagnosis, and treatment began in 1882 when German microbiologist Dr. Robert Koch identified *Mycobacterium tuberculosis* (*Mtb*) as the causative agent of TB (2). After entering the body through the respiratory system, *Mtb* is phagocytosed by alveolar macrophages, where it replicates within the phagosomes. As a protective response, immune cells surround the infected macrophages to contain the spread of the bacteria to other cells and thus contribute to the formation of granuloma (3). These granulomas are the result of an anti-TB immunological reaction, yet *Mtb* survives within the granuloma in a less metabolic state, called dormancy. However, upon immunosuppression, *Mtb* resuscitates into the replicative phase, leading to active pulmonary TB (PTB) and extra-pulmonary TB (EPTB) (4). As per WHO Report 2024, there were approximately 10.8 million TB patients and 1.25 million TB-related deaths in 2023 (5). It is appreciable to highlight that improvements in living conditions, sanitation, and the ongoing development of anti-TB medications led to a dramatic decline in TB incidence and mortality. However, the use of immunosuppressive drugs, drug addiction, poverty, population diversity, and the rise of drug-resistant strains of Mtb are still contributing to poor control of TB worldwide (6). Nearly 0.5 million Mtb patients have developed resistance to the front-line antibiotic rifampicin per year during the last five years, and about 80% of those individuals have multidrug resistance against TB. Thus, TB continues to pose a significant threat to public health worldwide.

Hence, an efficient vaccine is desperately needed for the long-term economic control of TB. The discovery of BCG was revolutionary in the field of vaccines. Developed over 13 years by Albert Calmette and Camille Guerin at the French Institute Pasteur, BCG is a live attenuated strain of *Mycobacterium bovis*, administered orally to infants as a vaccine against TB in 1921 (7). Since then, it has been the only licensed vaccine against TB with sub-optimal efficacy of 0-80% in adults.

Despite its use against TB, BCG is a broad-spectrum vaccine that imparts protection against several diseases including bladder cancer, fungal and parasitic infections (8). This trait is primarily considered to be provided by its ability to induce trained immunity. Trained immunity is a phenomenon of the generation of memory-like features in innate cells, majorly macrophages, dendritic cells, and NK cells. This process imprints certain epigenetic signatures in macrophages, that allow the synthesis and secretion of pro-inflammatory cytokines upon challenge with the unrelated pathogen. This mechanism has enabled the progress of BCG in protecting against various infections such as *Candida albicans*, bladder cancer, *S. aureus*, etc. Yet, BCG is less successful against TB and other diseases because of various host genetic factors such as genetic polymorphism, which contributes to variable responses to chemotherapy and vaccine efficacy (9). Significant genetic and allelic polymorphisms are the key attributes contributing to the variable efficacy of vaccines. Hence, it is imperative to consider these major determinants when designing a vaccine targeting the worldwide population. Thus, in this review, we aim to decipher the significance and mechanism of BCG-induced trained immunity. Despite BCG as the only vaccine against TB, we aim to shed light on the potential vaccine candidates that are currently under clinical trials. Lastly, we discuss the host genetic as the crucial factor that limits the efficacy of a single vaccine against TB in all ethnic regions. Vaccination aims to create a long-lasting immune memory for *Mtb* infection management. It triggers immune responses through various pathways, including B cells, CD4^+^ T cells, CD8^+^ T cells, and NK cells. CD4^+^ T cells develop into four primary subsets for TB protection: T central memory (T_CM_), T effector memory (T_EM_), T tissue-resident memory (T_RM_), and newly recruited T effector (T_EFF_) cells. Vaccines maintain a T_CM_ pool for long-term protection, but Mtb infection drives T cell differentiation towards late-stage T_EM_ and T_EFF_ responses (10).

BCG is traditionally administered intradermally to prime the immune system and generate protective responses against TB (11). It is phagocytosed by macrophages and dendritic cells, leading to increased expression of co-stimulatory molecules and MHC II (12). This results in enhanced cross-presentation of antigens to T cells, causing polarization of CD4^+^ T cells towards the T_H_1 phenotype and the secretion of pro-inflammatory cytokines (12). However, BCG fails to protect against PTB in adults. Efforts have been made to improve the efficacy of BCG, with different routes of administration being assessed. Intranasal and intravenous administration of BCG provides a rapid response in the lung, reducing Mtb burden more rapidly than intradermal BCG (8). However, BCG has sub-optimal protection against secondary infection, necessitating improvements in current BCG or the development of new therapeutics. Therefore, several candidates were developed in response to the demand for additional vaccinations to enhance BCG protection, which has been discussed below.

## 2 Current vaccine candidates in clinical trials

The three main pillars of the optimal TB vaccination strategy are the prevention of primary infection and disease following exposure, the avoidance of latent infection reactivation, and immunotherapeutic adjuvant to standard TB treatment for patient recovery (13). Inactivated vaccines live attenuated vaccines, recombinant BCG vaccines, subunit vaccines, viral vector vaccines, and DNA vaccines are among the novel TB vaccines presently undergoing clinical trials (14). Inactivated and Subunit vaccines are primarily utilized as therapeutic and prophylactic candidates against *Mtb*. Live attenuated vaccines are intended for the initial immunization of newborns or the prevention of TB in adolescents and adults. The list of vaccines undergoing clinical trials has been summarized in **Table 1**.

**Table 1:** TB vaccines in the preclinical or clinical stages of development.

Vaccine	Characteristics	Phase no.	Result	References
Inactivated				
MIP	Heat-inactivated whole cell vaccine	Phase III	The sputum culture conversion rate of the MIP group at week four was 67.1%, which was considerably greater than the 57% of the placebo group for category II TB patients.	(15)
SRL172	Derived from non-tuberculous mycobacteria	Phase III	When given to HIV-positive individuals who have received childhood BCG vaccination, a multiple-dose regimen of *Mycobacterium vaccae* is safe and significantly protective against TB.	(16)
RUTI^®^	Detoxified liposomal fragments of Mtb	Phase II	The RUTI^®^ vaccine developed particular humoral and cell-mediated immune responses in healthy individuals. The RUTI vaccine, despite its variable immunogenicity profile in LTBI subjects, is tolerable and could be considered for a single high-dose injection in future trials.	(17, 18)
Live attenuated				
MTBVAC	A mutant of Mtb that has deletion mutations in the virulence genes fadD26 and phoP, which encode two important virulence components.	Phase III	Safe and immunogenic in healthy adults. Although no statistical significance could be reached, the MTBVAC group had a higher frequency of polyfunctional CD4^+^ T_CM_ cells and more responders than the BCG group at the same dose. Produced a long-lasting CD4^+^ cell response in neonates and demonstrated adequate reactogenicity.	(19, 20)
BCG revaccination	BCG revaccination.	Phase IIb	Strong, multifunctional CD4^+^ T cells specific to BCG were produced by BCG revaccination in healthy individuals. BCG revaccination was immunogenic and decreased the rate of sustained QFT conversion in adolescents. The BCG-Denmark immunization in Brazilian healthcare professionals did not decrease the risk of QFT Plus conversion. BCG revaccination in HIV-negative adolescents without QFT tests did not offer protection against sustained Mtb infection. A Malawi study conducted on repeat BCG immunization without a placebo showed no significant protection against TB.	(21–25)
Recombinant BCG				
VPM1002	A recombinant BCG mutant that expresses listeriolysin O and has a deletion in urease C	Phase III	In both BCG-naïve and BCG-immune people, VPM1002 was safe and activated T cells that produced IFN-γ and B cells that produced antibodies. Safe, immunogenic, and immunologically tolerated in neonates. Produces less immune responses than BCG in HIV-exposed and unexposed infants	(26, 27)
Subunit				
M72/AS01E	M72 is an immunogenic fusion protein made from the adjuvant AS01E and two Mtb antigens (Mtb32A and Mtb39A).	Phase III	Adults receiving treatment for TB showed immunogenicity to the M72/AS01E vaccination. M72/AS01 was immunogenic and well tolerated in the group of ART-stable and ART-naïve, with HIV and without HIV adults. 54% of adults who received the M72/AS01E vaccine were protected against Mtb infection. M72/AS01E vaccine stimulated an immunological response and offered protection against the development of pulmonary TB illness for a minimum of 3 years.	(28–31)
GamTBvac	Includes the fusion proteins Ag85A and ESAT6-CFP10 and the adjuvant DEAE-dextran nanoparticle.	Phase III	In Mtb-uninfected volunteers who had previously had a BCG vaccination, the half-dose GamTBvac group demonstrated a strong, early, and sustained immune response. In healthy volunteers who had previously had a BCG vaccination, the GamTBvac vaccine produced antigen-specific interferon-gamma release, T_H_1 cytokine-expressing CD4^+^ T-cells, and IgG responses.	(32, 33)
H4:IC31 (AERAS-404)	Ag85B-TB10.4 fusion protein with IC31 adjuvant	Phase IIb	H4:IC31 showed a satisfactory safety profile and was immunogenic, eliciting multifunctional CD4^+^ T cell responses in healthy persons who had previously had a BCG vaccination. With an efficiency of 45.4%, the BCG vaccine decreased the rate of sustained QFT conversion; the H4:IC31 vaccine had an efficacy of 30.5%. The vaccination generated Ag85B-specific CD4^+^ T cell-mediated cellular immune responses and H4 antigen-specific IgG antibodies.	(24, 25, 34, 35)
ID93+GLA-SE	A recombinant fusion protein of Mtb antigens (virulence-associated Rv2608, Rv3619, Rv3620, and latency-associated Rv1813).	Phase IIa	A functional humoral and T_H_1 type cellular response is triggered by the GLA-SE adjuvant in BCG-naive, QuantiFERON-negative, healthy adults. Increasing doses of ID93+GLA-SE induced similar antigen-specific T cell and humoral responses in BCG-immunised, Mtb-infected individuals, with an acceptable safety profile, suggesting priming through natural infection. During the 6-month trial period, antigen-specific IgG and CD4^+^ T cell responses were considerably higher after two injections of GLA-SE than after receiving a placebo. In healthy adult healthcare workers who had received a BCG vaccination and were not infected with Mtb, the ID93 + GLA-SE vaccine produced antigen-specific cellular and humoral immune responses with a tolerable safety profile.	(36–39)
AEC/BC02	A recombinant vaccine expressing Ag85B and ESAT6-CFP10 fusion protein.	Phase IIa	Results awaited	
Viral vector				
MVA85A	Mtb Ag85A antigen-expressing modified Vaccinia Ankara virus	Phase IIb	Newborns exposed to HIV responded safely to the MVA85A prime immunization, which produced a mild antigen-specific immune response.	(36)
ChAdOx1.85A	A simian adenovirus expressing Ag85A.	Phase IIa	Good immunogenicity and tolerance in healthy people. Stronger BAL cellular responses were elicited by aerosol ChAdOx1-85A, particularly IFN-γ/IL17^+^CD4^+^ T cells. The Ag85A-specific IFN-γ in ChAdOx1 85A–MVA85A group response peaked on day 63 and was greater than in the BCG revaccination group.	(40–42)

### 2.1 Inactivated TB vaccines

To trigger an immune response against a range of *Mtb* antigens, inactivated vaccines employ either the whole or fragmented, lysed forms of *Mtb*. TB has traditionally been prevented and treated by inactivated vaccinations. These vaccines have demonstrated substantial immunotherapeutic benefits in TB control and elicit humoral and T_H_1 cell-mediated immune responses, protecting against extracellular *Mtb* infections (43). However, there are certain limitations associated with inactivated vaccines, such as the need for multiple doses and a shorter immunization period. Nonetheless, inactivated vaccines are rapidly evolving due to their advantages in production, stability, and safety (44). *Mycobacterium indicus pranii* (MIP), RUTI^®^, and SRL172 are the inactivated vaccines currently undergoing clinical trials.

#### 2.1.1 Mycobacterium indicus pranii

Mycobacterium w (*Mw*), also known as *Mycobacterium indicus pranii* (MIP), is a highly sought-after leprosy vaccine initially produced domestically in India (45). Initially characterized as a leprosy vaccine, genome screening revealed that MIP shares a vast repertoire of highly antigenic PE/PPE proteins with *Mtb* (46), indicating its potency as a TB vaccine candidate. While MIP administered subcutaneously diminishes the bacterial burden in the lungs, nasal administration of MIP further reduces the l burden and improves pulmonary pathology in mice (47). MIP induces autophagy, reversing phagosome maturation block and phagolysosome fusion inhibition, leading to enhanced *Mtb* clearance (48). TNF-α, IL-12p40, IL-6, nitric oxide, increased ROS, and IFN-induced chemokines like CXCL10 are among the pro-inflammatory reactions brought on by MIP (49). It triggers a T_H_1 and T_H_17 immune response generating IFN-γ^+^ TNF-α^+^ IL-2^+^ T cells, or IFN-γ^+^ TNF-α^+^ T cells (50), downregulates the T_H_2 pathway, and stimulates dendritic cells and macrophages (51).

As a potential TB vaccine, MIP is presently undergoing Phase III clinical studies. These studies are examining its effectiveness and safety in preventing TB, especially in healthy household contacts of TB patients. MIP was employed as a prophylactic vaccine against leprosy in a double-blind clinical experiment in household contacts of leprosy patients. A retrospective review conducted 13 years later showed that MIP provided considerable protection against TB as well, in an area where TB is very endemic and the subgroup that had BCG as a child had a much lower incidence of TB (52). The Ministry of Science and Technology, Government of India, and Cadila Pharmaceuticals Ltd., India, financed a phase III randomized, double-blind, placebo-controlled, multicenter clinical trial (NCT00265226) to assess the safety and effectiveness of MIP as a treatment for category II TB patients in India. Following four weeks of treatment, the MIP group’s sputum culture conversion rate (67.1%) was noticeably greater than the placebo group’s (57%), suggesting that MIP can eradicate bacteria (15). These findings show that MIP is safe and may help TB patients eliminate *Mtb* and highlight MIP’s potential as a TB vaccine that may be used both therapeutically and preventively, calling for more research into its modes of action and the best ways to administer it.

#### 2.1.2 SRL172

SRL172, an inactivated whole cell BCG booster made from a heat-inactivated non-tuberculous mycobacterium that was deposited at the National Collection of Type Cultures (NCTC, London, UK) under accession number 11659, was used by the Dartmouth group to start their investigations.

In Argentina, newly diagnosed PTB patients received triple-dose immunotherapy using SRL172 in conjunction with short-course, directly monitored chemotherapy. Compared to placebo receivers, immunotherapy recipients had a quicker recovery to normal values in all immunological markers (53). SRL172 has finished a Phase 3 clinical investigation demonstrating immunogenicity and efficacy in preventing culture-confirmed TB when tested on HIV-positive people in Tanzania (NCT02063555) (16). These features make SRL172 potent for TB vaccine development. To completely clarify the possible involvement of SRL172 in international TB prevention programs, more studies should concentrate on evaluating efficacy in various populations, examining long-term protection, and improving dose regimens.

#### 2.1.3 RUTI^®^

One of the few vaccines being developed exclusively as a therapeutic TB vaccine is the RUTI^®^ vaccine, which is presently in Phase III clinical trial. RUTI^®^ is a drug substance (DS) liposomal suspension that contains sucrose as a charge excipient and pure cellular fragments of Mtb bacilli grown under stress to simulate intra-granulomatous conditions. This confers the advantage of protecting against latent TB (54). Therefore, in experimental animal models of LTBI, the DS contains several antigens traditionally characterized as typical of replicating and non-replicating Mtb. RUTI^®^ causes a mixed T_H_1/T_H_2/T_H_3 (T_REG_) multi-antigenic immune response without causing systemic or local harm in animal models of TB (54) Compared to standard-of-care (SOC) chemotherapy, RUTI^®^ maintains the cellular immune response against ESAT-6 and dramatically lowers the bacterial burden in the lungs (55).

Phase I clinical trial (NCT00546273) in 2007 demonstrated the safety and immunogenicity of RUTI^®^ in healthy volunteers, showing that volunteers can develop specific humoral and cell-mediated immune responses to the RUTI^®^ vaccine (17). A phase II clinical trial of the RUTI^®^ vaccine (NCT01136161) was initiated in three South African regions in HIV-positive and negative LTBI individuals. The findings showed that while HIV-positive patients exhibited a similar poly antigenic immune response profile following the first vaccination, no significant increase in immune response was observed following the second vaccination. In comparison, HIV-negative patients who received RUTI^®^ showed good immune responses and a slight increase in immune response intensity after the second vaccination (18). These findings imply that the RUTI^®^ vaccine-induced immune responses vary across HIV-negative and positive populations, maybe as a result of the compromised CD4^+^ T cells in HIV-positive people. Two-phase IIb clinical trials (NCT04919239 and NCT05455112) are now seeking volunteers to assess the safety and efficacy of RUTI^®^ immunotherapy in TB patients in comparison to conventional treatments, as well as the efficacy of RUTI^®^ as an adjuvant for TB chemotherapy.

### 2.2 Attenuated TB vaccines

Attenuated TB vaccines serve various advantages over other vaccines, including their ability to elicit complex and varied immune responses. It can elicit immunological responses akin to those of a natural infection and can provide long-term protection. Attenuated vaccinations do, however, have certain disadvantages, one major being the possibility of regaining virulence Attenuated TB vaccines undergoing clinical studies include MTBVAC and BCG (revaccination).

#### 2.2.1 MTBVAC

The clinical strain Mtb Mt103 is a member of lineage 4 (Euro–African–American), one of the most prevalent lineages of Mtb, and contains most T cell epitopes specific to TB (56). MTBVAC is a live vaccine candidate rationally attenuated from this lineage. According to the Geneva Consensus guidelines for creating live-attenuated mycobacterial vaccines, MTBVAC attenuation is provided by two separate, unmarked deletions in the phoP and fadD26 pathogenicity genes. Studies on mice, guinea pigs, and rhesus macaques have shown that MTBVAC is safe, protective, and immunogenic (57, 58). Furthermore, co-administration of MTBVAC and BCG provides better protection in guinea pigs and adult and neonatal mouse models (59, 60).

MTBVAC reached clinical evaluation in newborns (NCT02729571) and adolescents (NCT02933281) in TB-endemic countries after completing the first-in-human phase 1 clinical trial for safety and immunogenicity in healthy adult volunteers in Switzerland in 2015 (19). Intradermal MTBVAC immunization has been shown to be acceptable reactogenicity and induced a durable CD4^+^ T-cell response similar to BCG in early-stage clinical studies in both adults and newborns (20). Two clinical trials, NCT02933281 and NCT03536117, are presently being conducted in South Africa to examine the many facets of MTBVAC. Additionally, MTBVAC (NCT04975178) is undergoing a randomized, double-blind, controlled phase III clinical trial in sub-Saharan Africa’s TB-endemic regions to assess its immunogenicity, safety, and effectiveness in infants with and without HIV exposure.

#### 2.2.2 BCG revaccination

Giving BCG again as a homologous boost is a clear way to increase its effectiveness. This approach is crucial because, should BCG revaccination enhance the protective efficacy of this century-old vaccine, we would have a low-cost, easily implementable instrument to aid in the improvement of TB control on a worldwide scale. The BCG vaccine has wide immunomodulatory effects on the innate and adaptive immune systems in addition to providing targeted protection against TB. In high-mortality areas, the BCG vaccine lowers infant mortality by over 40% and is used to treat malignancies including melanoma and bladder cancer (61, 62).

The current BCG vaccination schedule for infants is insufficient due to the lack of effective TB vaccine candidates. 78 nations, including China, advised giving the vaccine twice or more at varied intervals before 2019 (63). An alternate strategy using BCG prime-heterologous boosting regimens has emerged, but preclinical investigations have not shown enhanced efficacy against primary *Mtb* infection (64). Due to increased IL-2^+^ T_CM_ and IFN-γ^+^ T_EM_ cell responses after the booster vaccination, BCG-primed mice and then either revaccinated with BCG (65) or heterologous boosted with subunit protein CMFO vaccine are effective methods for preventing and controlling LTBI rather than primary Mtb infection (66). A study investigated the suboptimal efficacy of the BCG prime-boosting strategy against primary Mtb infection in C57BL/6J mice. Results showed that IL-10 is upregulated in the lungs of BCG-revaccinated mice, and blocking IL-10 signaling increases antigen-specific IFN-γ^+^ or IL-2^+^ CD4^+^ T cell responses and better protection against aerosol infection (67). BCG revaccination effectiveness against primary *Mtb* infection is less than 50%, with only individuals aged 31–40 showing protective effects. This necessitates more investigation into the ideal time frames between BCG vaccinations (68).

Phase 2b clinical trials are presently being conducted on BCG revaccination as a potential TB vaccine. A placebo-controlled trial of repeat BCG immunization in Malawi showed no significant protection against TB (21). A phase 2b trial found that BCG revaccination did not provide protection against sustained Mtb infection in HIV-negative adolescents (22). BCG revaccination has been employed in combination with other vaccine candidates which will be discussed in further sections.

A systematic review was conducted by Lawrence et al. to determine the effectiveness of BCG revaccination as a TB preventive measure. Studies and demographics differed in the protective efficacy of BCG revaccination; some showed only a moderate defense against the development of TB disease, especially in high-risk groups like healthcare professionals (69). A study conducted by Paulo dos Santos and colleagues found that neither the initial nor sustained QFT Plus conversion was shown to be protected by BCG revaccination; conversion was seen in 34 (3·4%) in the BCG group and 32 (3·2%) in the placebo group (23). Pre-sensitization with environmental mycobacteria, variations in BCG strain, administration site, and geographic location are all potential reasons for the disparities in protection offered by a single BCG therapy.

### 2.3 Recombinant BCG vaccines

Although the preventive effectiveness of BCG varies greatly among adults, other novel vaccinations cannot outperform the current BCG. Thus, one of the research avenues for TB vaccines is the appropriate recombination and modification of current BCG. The research techniques and outcomes of contemporary molecular biology, such as introducing exogenous target genes into pre-existing bacteria or viruses to employ them as carriers to create recombinant BCG (rBCG) vaccines, have greatly helped the study of BCG modification (70). Numerous forms of rBCG have been made, and human and animal models have been used to assess their protective benefits and humoral and cellular immune responses.

#### 2.3.1 VPM1002

The Max Planck Institute for Infectious Biology created the recombinant BCG vaccination VPM1002 (rBCG ΔUreC:: hly), which improves BCG immunogenicity by substituting the urease C encoding gene (UreC) with the Listeriolysin O (LLO)-producing gene of *Listeria monocytogenes* (Hly) (71). As a result of this alteration, LLO is secreted, facilitating the entry of DNA and antigens into the cytoplasm of the host cell for the MHC processing, triggering autophagy, inflammasome activation, and apoptosis. Early gene expression analysis in mice following VPM1002 immunization showed that similar to THP-1 cells, IL-18 and IL-1β expression was elevated, as was the expression of IFN-inducible genes like Tmem173 (STING), Gbps, and other GTPases (72). By day 14, the lymph nodes of mice immunized with VPM1002 showed higher levels of apoptosis than those of mice immunized with BCG (72). Since it takes 12–14 days for T cells to reach the lungs after Mtb infection, the lungs of the mice immunized with VPM1002 had more IL-2^+^TNF^+^ double cytokine generating cells seven days after Mtb challenge than animals that had BCG vaccination suggesting recall response (73). More T_CM_ and T_FH_ cells, higher T_H_17 responses, faster T cell migration to the lungs after *Mtb* challenge, and higher levels of anti-mycobacterial antibodies were all linked to the enhanced protection provided by VPM1002 immunization in the mouse model (72).

Phase III clinical studies are underway for VPM1002. To assess the safety and immunogenicity of VPM1002 in male human volunteers, a randomized, control, dose-escalating phase I clinical trial was conducted in Germany (NCT00749034). The findings showed that VPM1002 produces specific and dose-dependent immune responses (74). A follow-up phase II clinical trial (NCT01479972) was carried out in high-burden settings in South Africa in neonates who had not been exposed to HIV and had not received the BCG vaccination. After a single dose, the results showed that VPM1002 is safe, immunogenic, and immunologically tolerated in neonates (26). In a double-blind, randomized, active-controlled phase 2 trial (NCT02391415) in four South African health centers, the safety and immunogenicity of VPM1002 with BCG in newborns with and without HIV were assessed. Despite the immunogenic qualities of both vaccinations, VPM1002 produces fewer immune responses than BCG (27).

The small sample size of the aforementioned clinical studies is one of its limitations, making it challenging to create statistical comparisons of the immune responses induced by the two vaccines. Sample sizes have been raised to 2000 and 6940 participants in two recent phase III clinical trials (NCT03152903 and NCT04351685) that are still awaiting volunteer recruitment. These clinical trials aim to assess the safety and efficacy of VPM1002 in preventing TB infection in neonates and preventing TB recurrence in patients who have received successful treatment for TB.

### 2.4 Subunit TB vaccines

Proteins, peptides, amino acids, sugars, and other immunologically active substances separated and purified from *Mtb* comprise subunit TB vaccines. These vaccines have benefits, including effectiveness, safety, and affordability. However, their reduced antigen supply leads to a lesser immunological memory response and a weaker ability to trigger broad immunity. Adjuvants are, therefore, necessary for subunit vaccines to improve their immunogenicity, guarantee targeted distribution, and elicit immune protection. Following an initial BCG vaccination, they are frequently used as booster shots to increase BCG-mediated protection or prolong the duration of protection. To assess their effectiveness against *Mtb* infection or TB disease, clinical trials are presently being conducted for the five subunit vaccines for TB, including M72/AS01E, GamTBvac, H4: IC31 (AERAS-404), ID93+GLA-SE, and AEC/BC02.

#### 2.4.1 M72/AS01E

GlaxoSmithKline (GSK) in the UK created the subunit candidate TB vaccine M72/AS01E, which consists of the adjuvant AS01E and the highly immunogenic *Mtb* proteins *Mtb*39A and Mtb32A. M72/AS01E produced T_H_1-expressing, polyfunctional, M72-specific CD4^+^ and CD8^+^ T cells in mice (75). More fundamental research is required to fully comprehend the immune protection mechanisms of the M72/AS01E vaccination. Despite limitations related to the available animal studies, nonclinical evaluations (antigen-selection approach and *in vivo* preclinical data) and clinical safety and immunogenicity evidence, based on the capacity of the candidate vaccine responses, supported a proof-of-concept human trial (76).

The M72/AS01E TB vaccine candidate is currently in Phase 3 clinical trials, having been evaluated in a Phase 2b study. In individuals receiving treatment for TB, M72/AS01E produced strong M72-specific humoral and polyfunctional CD4^+^ T-cell mediated immune responses (28). The TB-treatment (adults receiving treatment for TB disease who have completed the intensive phase of treatment) and TB-treated (adults with a history of successfully treated pulmonary TB disease) groups had higher levels of polyfunctional M72-specific CD4^+^ T cells expressing CD40L^+^ TNF-α^+^ IFN-γ^+^, CD40L^+^ IL-2^+^ TNF-α^+^ IFN-γ^+^, or CD40L^+^ IFN-γ^+^ profiles than the TB-naïve (adults who have never had TB) group (28). HIV-positive and HIV-negative patients showed immunogenicity to M72/AS01E, with reactions persisting one year after vaccination. Polyfunctional CD4+ T-cell responses were produced seven days after administration (29). In a randomized, double-blind, placebo-controlled phase II clinical trial (NCT01755598) in Kenya, South Africa, and Zambia, the vaccine showed a 54% protective efficiency against *Mtb* infection in adults (30). The M72/AS01E immunization offered at least three years of immunological protection (49.7% protective efficacy) to prevent latent infections from becoming active TB cases (31). These studies show that M72/AS01E is immunogenic and safe for adults and adolescents.

Compared to a placebo, the two-dose M72/AS01E immunization increased seroconversion rates, according to a meta-analysis (77). M72/AS01E immunization produced strong, polyfunctional CD4+ T cells for three years, with TEM cells present and more memory TH1 cytokine-expressing responses than other novel vaccines (78). To assess the safety and immunogenicity of M72/AS01E in HIV-positive individuals receiving virus suppression and antiretroviral therapy, a randomized, placebo-controlled phase III clinical trial (NCT04556981) is presently underway in South Africa.

#### 2.4.2 GamTBvac

The recombinant subunit candidate vaccine GamTBvac was created and studied in response to the pressing need for TB vaccines around the world as well as to address the TB situation in Russia. GamTBvac is a recombinant vaccine that contains three Mtb antigens: Ag85a, CFP10, and ESAT6. These antigens are fused into two chimeric proteins from *Leuconostoc mesenteroides* that have a dextran-binding domain (DBD). These fusions were created using an adjuvant that contained CpG oligodeoxynucleotides (ODN), diethylaminoethyl (DEAE)-dextran 500 kDa, and dextran 500 kDa (79). GamTBvac showed substantial immunogenicity and notable protective benefits against the H37Rv strain in mice and guinea pigs TB models (80).

GamTBvac’s safety and immunogenicity in healthy persons who have received a BCG vaccination were assessed in a phase I clinical trial conducted in Russia. The half-dose vaccine group demonstrated the maximum immunogenicity (32). After that, 180 healthy volunteers who had not contracted Mtb and had received BCG vaccination participated in a double-blind, randomized, multicenter, placebo-controlled phase II clinical trial (NCT03878004). The vaccination produced substantial antigen-specific IFN-γ, T_H_1 cytokines, and IgG antibodies (33). A phase III clinical trial (NCT04975737) has been started to evaluate GamTBvac’s safety and effectiveness in preventing PTB in HIV-uninfected people between the ages of 18 and 45 in light of these encouraging findings.

#### 2.4.3 H4:IC31 (AERAS-404)

An immunological adjuvant known as IC31^®^ and the H4 antigen, a fusion protein made from two *Mtb*-antigens, Ag85B and TB10.4, make up the investigational vaccination H4:IC31 (AERAS-404). The rationale of the H4:IC31 vaccine is that by presenting *Mtb*-specific antigens in this context, T-cell immunity triggered by BCG may be enhanced, improving protection against TB. The leucine-rich peptide KLK and the synthetic oligonucleotide ODN1a combine to form the exclusive IC31 adjuvant (Valneva, Vienna, Austria).

A phase I trial of H4:IC31 in South African adults found it safe and immunogenic, with the 15 μg dose causing the most optimal immune response (34) In Switzerland, TB-negative, HIV-negative, BCG-unvaccinated adults (NCT02420444), TB-negative, HIV-negative, BCG-vaccinated adults (NCT02109874), HIV-negative, BCG-vaccinated adults in Sweden (NCT02066428) and Finland (NCT02074956), and HIV-uninfected, HIV-unexposed, BCG-primed infants in South Africa (NCT01861730) have all participated in several phase I clinical trials. Similar outcomes from these clinical trials have shown that H4:IC31 is safe for humans and triggers multifunctional CD4^+^ T_H_1 responses and IFN-γ production (35). A 2014 clinical trial in South Africa assessed the safety, immunogenicity, and prevention of *Mtb* infection with H4:IC31 and BCG revaccination in HIV-uninfected adolescents. Both vaccines were immunogenic, but H4:IC31 users had a poorer conversion rate (24). In Cape Town, South Africa, a phase 1b randomized clinical study (NCT02378207) was carried out in 2020 to assess the immunological responses and safety of three distinct vaccination regimens: BCG revaccination, H4:IC31, and H56:IC31. The data showed that the H4:IC31 and H56:IC31 vaccines produced Ag85B-specific CD4^+^ T cell-mediated cellular immunological responses and H4 and H56 antigen-specific IgG antibodies (25). Presently, H4:IC31 is in Phase II clinical trials assessing safety and immunogenicity in infants with BCG vaccination and ongoing in Africa.

#### 2.4.4 ID93+GLA-SE (QTP101)

Glucopyranosyl lipid adjuvant (GLA)-stable emulsion (SE) is a synthetic TLR 4 agonist that was created as an adjuvant with an oil-in-water emulsion; ID93 is made up of four *Mtb* antigens (Rv2608, Rv3619, Rv3620, and Rv1813) linked to virulence or latency (81). The production of T_H_1 CD4^+^ T cells by ID93/GLA-SE results in long-lasting immunity and inhibits pulmonary disease in C57BL/6 mice both early (4 weeks) and late (8 months) after challenge with *Mtb* HN878 (82). Several animal models have already shown the safety, immunogenicity, and pharmacodynamics of this vaccine (83).

To assess the safety and immunogenicity of two doses of ID93 antigen, either by itself or in adjunct with two doses of GLA-SE adjuvant, in healthy people, a phase I randomized, double-blind, dose-escalation clinical trial (NCT01599897) was carried out in the United States. The findings demonstrated that ID93, in adjunct with adjuvant GLA-SE, produced potent antibody and CD4^+^ T cell immunogenic responses (36). Similar results were obtained in follow-up phase I randomized, controlled, placebo-controlled, dose-escalation experiment (NCT01927159) carried out in South Africa in healthy HIV-negative people who have received the BCG vaccination (37) as well as in a phase IIa randomized, double-blind, placebo-controlled clinical trial (NCT02465216) conducted in Cape Town, South Africa, in adult TB patients who were HIV-uninfected following treatment (38). Furthermore, ID93+GLA-SE also made strong, long-lasting antibody responses and antigen-specific, multifunctional CD4^+^ T cell responses (38). A phase 2a trial in South Korea found that administering the ID93+GLA-SE vaccine to HIV-negative, BCG-vaccinated, and QuantiFERON-TB-negative adults significantly increased antigen-specific antibody levels and T_H_1 immune responses (39). All clinical trials of ID93+GLA-SE in various groups showed good safety and immunogenicity, which strongly supports the need for additional clinical trials. However, statistical differences cannot be reliably determined due to the small sample sizes in these investigations.

#### 2.4.5 AEC/BC02

Comprising the recombinant *Mtb* antigen Ag85b, ESAT6-CFP10, and a complex adjuvant system BC02, AEC/BC02 is a novel recombinant TB vaccine that has successfully finished phase I clinical trials (84). Combining three or six injections of the AEC/BC02 vaccine with four weeks of isoniazid-rifampin tablet treatment could improve the pathological lesions and drastically lower the bacterial load and gross pathological score in organs of a guinea pig latent infection model (85). These findings imply that AEC/BC02 might function as a therapeutic vaccination and stop latent infections from reactivating. AEC/BC02 vaccine immunotherapy significantly reduced bacterial loads in the lungs and spleen of mice, possibly related to the antigen-specific IFN-γ and IL-2 cellular immune responses induced by AEC/BC02 (86). This study also confirmed the therapeutic effect of the AEC/BC02 vaccine on latent infection with *Mtb* in mice.

Consequently, Anhui Zhifei Longcom Biopharmaceutical Co., Ltd. launched a phase I clinical research (NCT03026972) in 2017 to assess the safety of the AEC/BC02 vaccine. Additionally, a single-center, single-dose, placebo-controlled Phase Ib clinical trial (NCT04239313) was subsequently carried out in 2020 in healthy individuals. Although participant enrollment for both clinical trials is complete, the results have not been published. To assess the safety, tolerability, and immunogenicity of the AEC/BC02 vaccine in LTBI patients aged 18 and older, a phase II double-blind, randomized, controlled clinical trial (NCT05284812) was started in Hunan, China, in March 2022. Volunteers for the trial are currently being recruited. This study will offer more clinical proof of the effectiveness of AEC/BC02 as a possible TB vaccine.

### 2.5 Viral vector-based TB vaccines

Viral vector vaccines are platforms made to overexpress antigens and trigger immune responses without needing an adjuvant (87). The following are some of the benefits of viral vector vaccines (1): they can carry genes that encode large antigenic regions (2); they can elicit high levels of humoral and cellular immune responses; and (3) they don’t need adjuvants to boost their immune response. Nevertheless, there are also disadvantages, like the resurgence of virulence, erratic exogenous gene expression, or possible contribution of induced or pre-existing anti-vector immunity. Modified vaccinia virus Ankara (MVA), influenza, hemagglutinating, adenovirus Ad5 and Ad35, Sendai, and simian adenovirus are common viral vectors utilized in developing TB vaccines. Clinical trials for TB are presently being conducted on viral vector-based vaccines, such as MVA85A, ChAdOx1.85A, TB/FLU-01L, TB/FLU-04L, and AdHu5Ag85A.

#### 2.5.1 MVA85A

MVA85A, the most clinically progressed novel vaccine candidate, is a recombinant replication-deficient MVA that expresses the immunodominant *Mtb* Ag85A. The BCG-MVA85A vaccination program can protect against *Mtb* in guinea pigs (88), NHPs (89), and cattle (90). It also produces high cellular immunity in mice and calves (90, 91).

Phase II clinical studies are presently being conducted on MVA85A. A phase I clinical trial (NCT00460590) was carried out in Cape Town, South Africa, in 2007 to assess the immunogenicity and safety of MVA85A in healthy participants. The findings demonstrated that the vaccination produced strong, long-lasting, and antigen-specific CD4^+^ T cell responses (92). A phase II clinical trial (NCT00679159) was carried out in 2014 in healthy infants and children who had received the BCG vaccination in South Africa. The findings, which were released seven years later, indicated that the MVA85A vaccination produced strong, long-lasting, polyfunctional CD4^+^ and CD8^+^ T cell responses in newborns (93). A randomized trial compared MVA85A prime vaccination to Candin^®^ control and BCG vaccination for HIV-uninfected infants. MVA85A vaccination induced higher Ag85A-specific IFN-γ^+^ CD4^+^ T cells than control, but BCG did not further boost this response. BCG-induced Ag85A-specific IFN-γ^+^ CD4^+^ T-cell response was similar across study arms (68). Although the precise mechanism behind MVA85A’s protective efficacy is unknown, its low antigen complexity is thought to be the reason for its clinical failure, which led to the development of many vaccine candidates with diverse antigens.

#### 2.5.2 ChAdOx1.85A

Live, non-replicating viral vector-based vaccines are safe and efficient (94). A new chimpanzee adenoviral vector vaccination that expresses Ag85A is ChAdOx1.85A. Both T cell and B cell responses were elicited by a combination of viral-vectored vaccines that expressed the immunogenic and immunodominant secretory TB Ag85a (ChAdOx1 85A prime, followed by MVA85A boost [ChAdOx1 85A–MVA85A]). In a mouse model, this combination of vaccines was more effective than BCG vaccination alone (95).

In healthy people in the UK, a Phase I clinical trial assessed the safety and immunogenicity of the ChAdOx1.85A prime-MVA85A boost vaccine, showing good immunogenicity and tolerance (40). The proposed TB vaccine ChAdOx1-85A was administered by aerosol versus intramuscular route in a randomized, double-blind, controlled phase 1 experiment (SNCTP000002920). The intramuscular ChAdOx1-85A vaccination produced stronger humoral and systemic cellular responses, while the aerosol ChAdOx1-85A vaccination produced stronger BAL cellular responses, especially IFN-γ/IL17^+^CD4^+^ T cells (41). The viral vector intramuscular vaccination ChAdOx1.85A + MVA85A finished its phase IIa clinical study in 60 adolescents. Compared to the BCG vaccine, the vaccine produced greater Ag85A-specific responses (42).

## 3 Host genetics play a role in vaccine efficacy

The most crucial step in the fight against infectious diseases is vaccination. Indirect protection, in which other people are immunized to lessen transmission, and direct protection, in which high-risk individuals are vaccinated to prevent the disease, are the two ways that an effective vaccination can protect people (96). Despite numerous advancements in vaccination research, doubts remain over the effectiveness and longevity of vaccines.

The effectiveness of vaccines differs in individuals and populations. Individual differences in antibody responses to the hepatitis B vaccine and yellow fever vaccine are more than 10 and 100 times, respectively. The protective effect and length of the vaccine’s immunity are affected by these differences in the vaccination response. It’s concerning that some vaccinated individuals develop disease. This indicates that the vaccine is ineffective in certain individuals (non-responders). The nature of the infectious agent (genetic variation), vaccine factors (vaccine type, adjuvant, dose, and administration route and schedule), and host factors (age, sex, genetics, nutritional status, gut microbiota, obesity, and immune history) all affects the effectiveness of a vaccine (97). Considering all these factors is beyond the scope of this review; however, the review aims to shed light on one of the important factors, i.e., host genetics, which is considered to be responsible for the variable efficacy of the BCG vaccine.

**Figure 1:**
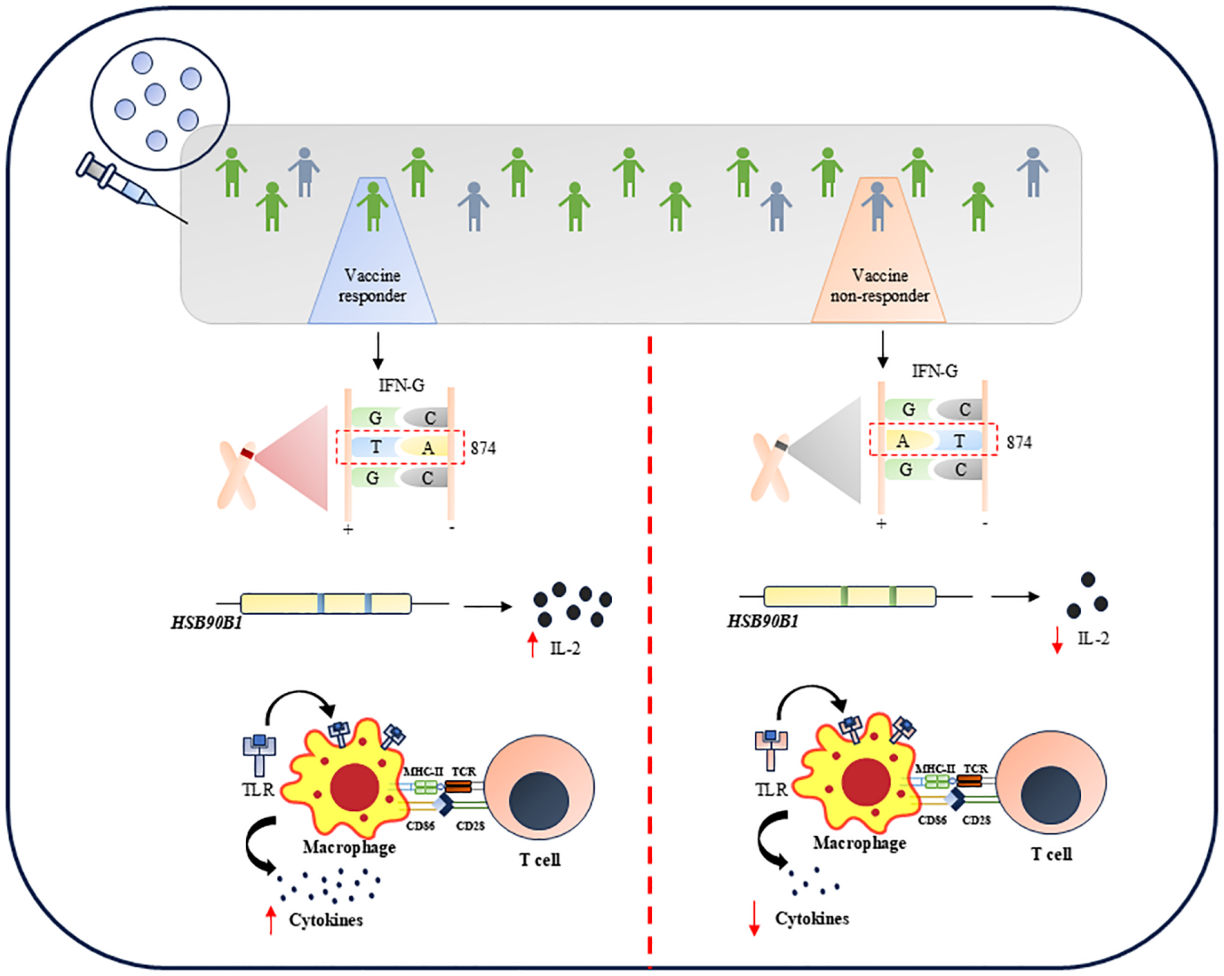
Several genetic variations offers limited vaccination response against TB.

Genetic background has a profound effect on the disease outcome. The plethora of reports mention that population genetic diversity determines the severity of the disease and hence the treatment efficacy (98–100). This variability of BCG in protecting against TB is highly prominent due to population and genetic diversity (101). Thus, understanding the host factors such as allelic polymorphism and single nucleotide polymorphism is important to develop a vaccine with higher protective efficiency against a wide, diverse population. Limited studies have shown subtle variations at the genetic level in certain immune factors, as depicted in **Figure 1** and as discussed below.

### 3.1 Genetic variation in interferon

IFN-γ is a potential marker associated with the anti-mycobacterial response. The amplitude of the post-vaccination IFN-γ response may be influenced by the genetic background of those who received the BCG vaccination. One of the most well-studied polymorphisms influencing IFN-γ production is the T>A single nucleotide polymorphism (SNP) of *IFN-G* in the +874 position (102). It has been linked to the development of active TB (102), as well as to decrease IFN-γ production in TB patients (103) and in children who have received the BCG vaccine. Anuradha et al., in 2008, demonstrated that IFN-γ (+874T/A) polymorphism is the independent factor that determines the vaccine outcome in both scar-positive and scar-negative children who received BCG (104). This study remarkably showed that children with TT genotype in the IFN-G gene produce more IFN-γ while those with AT or AA genotype secreted less IFN-γ and hence were more susceptible to active TB. It was also shown that children with TT genotype have BCG scars corresponding to better immune response (104). Conversely, there are two schools of thought suggesting the influence of genetic variation in interferon genes on BCG efficacy. In 2019, a report showed that any genetic variation in IFN-γ, specifically rs2430561 and rs35314021, did not significantly hamper BCG efficacy. However, the studies carried out to undermine the importance of genetic variation in IFN-γ were based on different BCG strains, which may contribute as confounders to the experimental data shown (105). Further, high-density genotyping and imputation showed that ~100,000 variants are associated with IFN-γ production in TB households in France and South Africa (106). Among them, the most prominent genetic variation was rs9828868 on chromosome 3q, an expressive quantitative trait loci (eQTL) of the ZXDC gene. This gene is linked to IL-12 production through CCL2/MCP1. Thus, the study highlights the significant association of the genetic variant rs9828868 with IFN-γ production in response to *Mtb*, emphasizing its potential role in understanding human immunity against TB and the genetic factors that contribute to variability in immune responses.

### 3.2 Genetic variation in TLR signaling pathway

Autophagy (107), endosomal transport (108), TLR signaling (109), and the activation of IL-1β, other cytokines, and antimicrobial compounds (110) are some of the variables that affect the development of effective immune responses to *Mtb*. Many of these vital immunological functions, including TLR2, TLR4, IL-1R signaling, autophagy, and endosomal transport, are regulated by toll-interacting protein (TOLLIP). Additionally, there is a substantial correlation between TOLLIP variation and adult TB disease risk (111). BCG responses are also linked to two SNPs in the HSP90B1 gene, which codes for a TLR protein chaperone (112). These SNPs’ minor alleles are linked to enhanced *in vitro* IL-2 responses and defense against juvenile TB.

To comprehend how TOLLIP modifies human immune function following mycobacterial infection, Shah et al., 2017 identified a common functional variation of the TOLLIP gene. Single nucleotide polymorphisms in innate and adaptive immune-related genes alter BCG-induced responses, such as those in the TLR 2, 6, 10, and TOLLIP genes, which affect BCG-induced cytokine secretion (113). The efficacy of the vaccine also differs with the magnitude of the response to the vaccine elicited by the host. This is evident from a study conducted in 2018 which revealed that compared to non-reactogenic strains, mice vaccinated with MTBVAC and reactogenic for CFP10 and ESAT6 are more protected against TB (114). About 60–80% of people in humans are responders to these antigens (115). Compared to BCG, a vaccination that expresses ESAT6 and CFP10 would provide these people with better protection; however, further clinical evidence would be required to verify this theory (115).

Since BCG is a live attenuated strain of *Mycobacterium bovis*, developing a BCG infection—which can be systemic (like BCG osteitis, disseminated BCG illness) or regional (like BCG lymphadenitis)—is one of the major side effects of BCG vaccination. *In vitro*, BCG-induced IFN-γ and IL-2 were previously linked to three of the four SNPs in TLR genes in samples from healthy newborns who received the BCG vaccination (116). In samples from BCG osteitis survivors, only the TLR2 SNP +2258G > A (rs5743708) was linked to altered cytokine responses (117). According to these findings, those who have particular SNPs in the cytokine and TLR genes may be more susceptible to adverse side effects from the BCG vaccination.

### 3.3 Experimental animals for studying the effects of genetic variation in hosts

The varying effectiveness of TB vaccinations reflects the variability in TB susceptibility. Studies conducted in various geographical locations and ethnic groups over the ensuing decades have shown wildly disparate estimations of the impact of BCG. Numerous studies have examined the functions of genetic variation in the vaccine strain (118) and prior exposure to environmental mycobacteria (119). However, it has proven more challenging to measure the contribution of host genetic diversity to BCG efficacy. Variations in a large number of immune mediators are highly heritable (120). The number of T_CM_ cells or the number of cytokines like IL-12p40, granulocyte-macrophage colony-stimulating factor (GM-CSF), IFN-α, and IL-6 are among the mediators that are likely to be important to *Mtb* immunity that is impacted by many of these heritable variations (121). In fact, a number of studies indicate that the immune response to BCG vaccination (116) or mycobacterial infection (122) is heritable.

The genetic determinants of vaccination protection and TB susceptibility may be unraveled using traceable model systems that include pertinent genetic variation. Smith et al. created a model system that captures the range of immunological responses seen in outbred individual mice, which can be used to comprehend the contribution of host genetics to vaccine efficacy (123) A panel of extremely varied inbred mouse strains, including the founders and recombinant offspring of the “Collaborative Cross” project, were used in this system, similar to natural populations in a number of significant ways: the animals showed a wide range of susceptibility to *Mtb*, varied in how they responded to infection immunologically and were not long-term protected by BCG vaccination. Vaccination only provided protection for a small percentage of the genotypes. These findings provide credence to the idea that host genetic variability plays a significant role in determining the effectiveness of BCG and offers a fresh tool for the logical development of more widely effective vaccinations.

Future research should concentrate on the following areas to address these challenges (1): conducting extensive clinical trials in a variety of populations to evaluate the effectiveness of vaccines across genetic backgrounds and environmental conditions (2); creating customized vaccination plans that consider individual genetic and environmental factors (3); examining the effects of co-infections and nutritional status on vaccine efficacy and figuring out how to optimize vaccination in these situations; (4) putting in place post-vaccination surveillance programs to track long-term efficacy and identify potential factors affecting vaccine performance in various populations; (5) designing targeted vaccines for particular population groups based on their *Mtb* infection status (LTBI, active TB, subclinical TB, or uninfected) (124).

There is much promise for TB prevention and treatment with precision medicine. This method customizes therapy regimens based on the patient’s unique genetic background, immunological condition, and disease phenotype (1) Genotype-Based Treatment: The most suitable medications and dosages can be chosen by predicting the responses of the patient to particular medications based on their genotype. For example, some genetic variants may affect how drugs are metabolized, which could lead to either increased or decreased pharmacological efficacy. By recognizing these differences, individualized treatment plans are possible. (2) Immune Status Monitoring: Personalized medicine can also help develop TB vaccines. The best time and amount to administer a vaccination can be established by tracking the immunological status of the patient. Deep learning technologies, for instance, can optimize vaccination tactics by analyzing the immunological state to forecast the time and intensity of immune responses. (3) Therapeutic vaccinations: A promising approach to treating patients who have already contracted *Mtb* is therapeutic vaccinations. These vaccinations usually target particular bacterial antigens to stimulate the immune system of the patient and aid in clearing the infection. For instance, following brief chemotherapy, the RUTI^®^ vaccine, a non-live multi-antigen vaccine derived from broken bacterial cell walls, has demonstrated promise in reducing LTBI (125).

## Concluding remarks

BCG harbors various immunodominant antigens and hence protects against various diseases through trained immunity. However, BCG serves sub-optimal protection against TB. Thus, extensive efforts are put in to either improve BCG or develop a new vaccine against TB. Nevertheless, merely complementing the vaccine candidate with *Mtb* antigens will not solve the purpose of enhancing vaccine efficacy. Through this review, we aim to highlight the potential efforts made to improve or develop the TB vaccine. Yet, there are other crucial factors, such as host responses, that are fundamental to the protective efficiency of any vaccine and thus should be captured for designing new therapeutics. Thus, it is necessary to revise the strategy for vaccine design so as to induce higher protection against TB worldwide.

## References

[1] Cucinotta D Vanelli M. WHO declares COVID-19 a pandemic. Acta Biomed. (2020) 91:157–60. Available online at: https://www.mattioli1885journals.com/index.php/actabiomedica/article/view/9397 (Accessed February 1, 2025). 10.23750/abm.v91i1.9397 PMC7569573 32191675

[2] Barberis I Bragazzi NL Galluzzo L Martini M. The history of tuberculosis: from the first historical records to the isolation of Koch’s bacillus. J Prev Med Hyg. (2017) 58:E9. Available online at: https://pmc.ncbi.nlm.nih.gov/articles/PMC5432783/., PMID: 28515626 PMC5432783

[3] Flynn JL Chan J Lin PL. Macrophages and control of granulomatous inflammation in tuberculosis. Mucosal Immunol. (2011) 4:271–8. doi: 10.1038/mi.2011.14, PMID: 21430653 PMC3311958

[4] Jellinger KA Bustin S Alsayed SSR Gunosewoyo H. Tuberculosis: pathogenesis, current treatment regimens and new drug targets. Int J Mol Sci. (2023) 24:5202. Available online at: https://www.mdpi.com/1422-0067/24/6/5202/htm (Accessed February 16, 2025)., PMID: 36982277 10.3390/ijms24065202 PMC10049048

[5] Floyd K Glaziou P Houben RMGJ Sumner T White RG Raviglione M. Global tuberculosis targets and milestones set for 2016-2035: Definition and rationale. Int J Tuberculosis Lung Disease. (2018) 22:723–30. doi: 10.5588/ijtld.17.0835, PMID: 29914597 PMC6005124

[6] Organization WHealth. Global tuberculosis report 2013. Geneva Switzerland: WHO (2013) 303.

[7] Fritschi N Curtis N Ritz N. Bacille Calmette Guérin (BCG) and new TB vaccines: Specific, cross-mycobacterial and off-target effects. Paediatr Respir Rev. (2020) 36:57–64. doi: 10.1016/j.prrv.2020.08.004, PMID: 32958428 PMC7439992

[8] Singh S Saavedra-Avila NA Tiwari S Porcelli SA. A century of BCG vaccination: Immune mechanisms, animal models, non-traditional routes and implications for COVID-19. Front Immunol. (2022) 13:959656. doi: 10.3389/fimmu.2022.959656, PMID: 36091032 PMC9459386

[9] Kumar P. A perspective on the success and failure of BCG. Front Immunol. (2021) 12:778028. Available online at: https://pmc.ncbi.nlm.nih.gov/articles/PMC8712472/. 34970263 10.3389/fimmu.2021.778028 PMC8712472

[10] Andersen P Scriba TJ. Moving tuberculosis vaccines from theory to practice. Nat Rev Immunol. (2019) 19:9. Available online at: https://www.nature.com/articles/s41577-019-0174-z (Accessed January 26, 2025)., PMID: 31114037 10.1038/s41577-019-0174-z

[11] Bollampalli VP Harumi Yamashiro L Feng X Bierschenk D Gao Y Blom H. BCG skin infection triggers IL-1R-myD88-dependent migration of epCAMlow CD11bhigh skin dendritic cells to draining lymph node during CD4+ T-cell priming. PloS Pathog. (2015) 11:e1005206. doi: 10.1371/journal.ppat.1005206, PMID: 26440518 PMC4594926

[12] Covián C Fernández-Fierro A Retamal-Díaz A Díaz FE Vasquez AE Lay MK. BCG-induced cross-protection and development of trained immunity: implication for vaccine design. Front Immunol. (2019) 10:2806. Available online at: https://pmc.ncbi.nlm.nih.gov/articles/PMC6896902/., PMID: 31849980 10.3389/fimmu.2019.02806 PMC6896902

[13] Ginsberg AM. What’s new in tuberculosis vaccines? Bull World Health Organ. (2002 [) 80:483. Available online at: https://pmc.ncbi.nlm.nih.gov/articles/PMC2567546/., PMID: 12132007 PMC2567546

[14] Cable J Srikantiah P Crowe JE Pulendran B Hill A Ginsberg A. Vaccine innovations for emerging infectious diseases—a symposium report. Ann N Y Acad Sci. (2020) 1462:14–26. doi: 10.1111/nyas.14235, PMID: 31659752

[15] Sharma SK Katoch K Sarin R Balambal R Kumar Jain N Patel N. Efficacy and Safety of Mycobacterium indicus pranii as an adjunct therapy in Category II pulmonary tuberculosis in a randomized trial. Sci Rep. (2017) 7:1. Available online at: https://www.nature.com/articles/s41598-017-03514-1 (Accessed March 6, 2025)., PMID: 28611374 10.1038/s41598-017-03514-1 PMC5469738

[16] Von Reyn CF Lahey T Arbeit RD Landry B Kailani L Adams LV. Safety and immunogenicity of an inactivated whole cell tuberculosis vaccine booster in adults primed with BCG: A randomized, controlled trial of DAR-901. PloS One. (2017) 12:e0175215. doi: 10.1371/journal.pone.0175215, PMID: 28498853 PMC5429024

[17] Vilaplana C Montané E Pinto S Barriocanal AM Domenech G Torres F. Double-blind, randomized, placebo-controlled Phase I Clinical Trial of the therapeutical antituberculous vaccine RUTI^®^ . Vaccine. (2010) 28:1106–16. doi: 10.1016/j.vaccine.2009.09.134, PMID: 19853680

[18] Nell AS D’Lom E Bouic P Sabaté M Bosser R Picas J. Safety, tolerability, and immunogenicity of the novel antituberculous vaccine RUTI: randomized, placebo-controlled phase II clinical trial in patients with latent tuberculosis infection. PloS One. (2014) 9:e89612. doi: 10.1371/journal.pone.0089612, PMID: 24586912 PMC3935928

[19] Spertini F Audran R Chakour R Karoui O Steiner-Monard V Thierry AC. afety of human immunisation with a live-attenuated Mycobacterium tuberculosis vaccine: A randomised, double-blind, controlled phase I trial. Lancet Respir Med. (2015) 3:953–62. Available online at: https://www.thelancet.com/action/showFullText?pii=S221326001500435X (Accessed March 6, 2025)., PMID: 26598141 10.1016/S2213-2600(15)00435-X

[20] Tameris M Mearns H Penn-Nicholson A Gregg Y Bilek N Mabwe S. Live-attenuated Mycobacterium tuberculosis vaccine MTBVAC versus BCG in adults and neonates: a randomised controlled, double-blind dose-escalation trial. Lancet Respir Med. (2019) 7:757–70. Available online at: https://www.thelancet.com/action/showFullText?pii=S2213260019302516 (Accessed March 6, 2025)., PMID: 31416768 10.1016/S2213-2600(19)30251-6

[21] Glynn JR Fielding K Mzembe T Sichali L Banda L McLean E. BCG re-vaccination in Malawi: 30-year follow-up of a large, randomised, double-blind, placebo-controlled trial. Lancet Glob Health. (2021) 9:e1451–9. Available online at: https://www.thelancet.com/action/showFullText?pii=S2214109X21003090 (Accessed May 25, 2025)., PMID: 34534489 10.1016/S2214-109X(21)00309-0 PMC8459381

[22] Schmidt AC Fairlie L Hellström E Kany ALK Middelkoop K Naidoo K. BCG revaccination for the prevention of mycobacterium tuberculosis infection. New Engl J Med. (2025) 392:1789–800. doi: 10.1056/NEJMoa2412381, PMID: 40334156 PMC12061034

[23] dos Santos PCP Messina NL de Oliveira RD da Silva PV Puga MAM Dalcolmo M. Effect of BCG vaccination against Mycobacterium tuberculosis infection in adult Brazilian health-care workers: a nested clinical trial. Lancet Infect Dis. (2024) 24:594–601. Available online at: https://www.thelancet.com/action/showFullText?pii=S1473309923008186 (Accessed March 14, 2025)., PMID: 38423021 10.1016/S1473-3099(23)00818-6 PMC11111441

[24] Nemes E Geldenhuys H Rozot V Rutkowski KT Ratangee F Bilek N. Prevention of M. tuberculosis infection with H4:IC31 vaccine or BCG revaccination. New Engl J Med. (. 2018) 379:138–49. doi: 10.1056/NEJMoa1714021, PMID: 29996082 PMC5937161

[25] Bekker LG Dintwe O Fiore-Gartland A Middelkoop K Hutter J Williams A. A phase 1b randomized study of the safety and immunological responses to vaccination with H4:IC31, H56:IC31, and BCG revaccination in Mycobacterium tuberculosis-uninfected adolescents in Cape Town, South Africa. EClinicalMedicine. (2020) 21:100313. Available online at: https://www.thelancet.com/action/showFullText?pii=S2589537020300572 (Accessed March 7, 2025)., PMID: 32382714 10.1016/j.eclinm.2020.100313 PMC7201034

[26] Loxton AG Knaul JK Grode L Gutschmidt A Meller C Eisele B. Safety and immunogenicity of the recombinant mycobacterium bovis BCG vaccine VPM1002 in HIV-unexposed newborn infants in South Africa. Clin Vaccine Immunol. (2017) 24:e00439-16. doi: 10.1128/CVI.00439-16, PMID: 27974398 PMC5299117

[27] Cotton MF Madhi SA Luabeya AK Tameris M Hesseling AC Shenje J. Safety and immunogenicity of VPM1002 versus BCG in South African newborn babies: a randomised, phase 2 non-inferiority double-blind controlled trial. Lancet Infect Dis. (2022) 22:1472–83. doi: 10.1016/s1473-3099(22)00222-5, PMID: 35772447

[28] Gillard P Yang PC Danilovits M Su WJ Cheng SL Pehme L. Safety and immunogenicity of the M72/AS01E candidate tuberculosis vaccine in adults with tuberculosis: A phase II randomised study. Tuberculosis. (2016) 100:118–27. doi: 10.1016/j.tube.2016.07.005, PMID: 27553419

[29] Kumarasamy N Poongulali S Bollaerts A Moris P Beulah FE Ayuk LN. A randomized, controlled safety, and immunogenicity trial of the M72/AS01 candidate tuberculosis vaccine in HIV-positive Indian adults. Medicine. (2016) 95:e2459. Available online at: https://journals.lww.com/md-journal/fulltext/2016/01190/a_randomized,_controlled_safety,_and.20.aspx (Accessed March 12, 2025)., PMID: 26817879 10.1097/MD.0000000000002459 PMC4998253

[30] Van Der Meeren O Hatherill M Nduba V Wilkinson RJ Muyoyeta M Van Brakel E. Phase 2b controlled trial of M72/AS01 E vaccine to prevent tuberculosis. New Engl J Med. (2018) 379:1621–34. doi: 10.1056/NEJMoa1803484, PMID: 30280651 PMC6151253

[31] Tait DR Hatherill M van der Meeren O Ginsberg AM Van Brakel E Salaun B. Final analysis of a trial of M72/AS01 E vaccine to prevent tuberculosis. New Engl J Med. (2019) 381:2429–39. doi: 10.1056/nejmoa1909953, PMID: 31661198

[32] Vasina DV Kleymenov DA Manuylov VA Mazunina EP Koptev EY Tukhovskaya EA. First-in-human trials of gamTBvac, a recombinant subunit tuberculosis vaccine candidate: safety and immunogenicity assessment. Vaccines. (2019) 7:166. Available online at: https://www.mdpi.com/2076-393X/7/4/166/htm (Accessed March 8, 2025)., PMID: 31683812 10.3390/vaccines7040166 PMC6963980

[33] Tkachuk AP Bykonia EN Popova LI Kleymenov DA Semashko MA Chulanov VP. Safety and immunogenicity of the gamTBvac, the recombinant subunit tuberculosis vaccine candidate: A phase II, multi-center, double-blind, randomized, placebo-controlled study. Vaccines. (2020) 8:652. Available online at: https://www.mdpi.com/2076-393X/8/4/652/htm (Accessed March 8, 2025)., PMID: 33153191 10.3390/vaccines8040652 PMC7712213

[34] Geldenhuys H Mearns H Miles DJC Tameris M Hokey D Shi Z. The tuberculosis vaccine H4:IC31 is safe and induces a persistent polyfunctional CD4 T cell response in South African adults: A randomized controlled trial. Vaccine. (2015) 33:3592–9. doi: 10.1016/j.vaccine.2015.05.036, PMID: 26048780

[35] Norrby M Vesikari T Lindqvist L Maeurer M Ahmed R Mahdavifar S. Safety and immunogenicity of the novel H4:IC31 tuberculosis vaccine candidate in BCG-vaccinated adults: Two phase I dose escalation trials. Vaccine. (2017) 35:1652–61. doi: 10.1016/j.vaccine.2017.01.055, PMID: 28216183

[36] Coler RN Day TA Ellis R Piazza FM Beckmann AM Vergara J. The TLR-4 agonist adjuvant, GLA-SE, improves magnitude and quality of immune responses elicited by the ID93 tuberculosis vaccine: first-in-human trial. NPJ Vaccines. (2018) 3:1. Available online at: https://www.nature.com/articles/s41541-018-0057-5 (Accessed March 2, 2025)., PMID: 30210819 10.1038/s41541-018-0057-5 PMC6123489

[37] Penn-Nicholson A Tameris M Smit E Day TA Musvosvi M Jayashankar L. Safety and immunogenicity of the novel tuberculosis vaccine ID93 + GLA-SE in BCG-vaccinated healthy adults in South Africa: a randomised, double-blind, placebo-controlled phase 1 trial. Lancet Respir Med. (2018) 6:287–98. Available online at: https://www.thelancet.com/action/showFullText?pii=S2213260018300778 (Accessed March 8, 2025)., PMID: 29595510 10.1016/S2213-2600(18)30077-8

[38] Day TA Penn-Nicholson A Luabeya AKK Fiore-Gartland A Du Plessis N Loxton AG. Safety and immunogenicity of the adjunct therapeutic vaccine ID93 + GLA-SE in adults who have completed treatment for tuberculosis: a randomised, double-blind, placebo-controlled, phase 2a trial. . Lancet Respir Med. (2021) 9:373–86. Available online at: https://www.thelancet.com/action/showFullText?pii=S2213260020303192 (Accessed March 8, 2025)., PMID: 33306991 10.1016/S2213-2600(20)30319-2

[39] Choi YH Kang YA Park KJ Choi JC Cho KG Ko DY. Safety and immunogenicity of the ID93 + GLA-SE tuberculosis vaccine in BCG-vaccinated healthy adults: A randomized, double-blind, placebo-controlled phase 2 trial. Infect Dis Ther. (2023) 12:1605. Available online at: https://pmc.ncbi.nlm.nih.gov/articles/PMC10173211/., PMID: 37166567 10.1007/s40121-023-00806-0 PMC10173211

[40] Wilkie M Satti I Minhinnick A Harris S Riste M Ramon RL. A phase I trial evaluating the safety and immunogenicity of a candidate tuberculosis vaccination regimen, ChAdOx1 85A prime – MVA85A boost in healthy UK adults. Vaccine. (2020) 38:779–89. doi: 10.1016/j.vaccine.2019.10.102, PMID: 31735500 PMC6985898

[41] Audran R Karoui O Donnet L Soumas V Fares F Lovis A. Randomised, double-blind, controlled phase 1 trial of the candidate tuberculosis vaccine ChAdOx1-85A delivered by aerosol versus intramuscular route. J Infection. (2024) 89:106205. doi: 10.1016/j.jinf.2024.106205, PMID: 38897242

[42] Wajja A Nassanga B Natukunda A Serubanja J Tumusiime J Akurut H. Safety and immunogenicity of ChAdOx1 85A prime followed by MVA85A boost compared with BCG revaccination among Ugandan adolescents who received BCG at birth: a randomised, open-label trial. Lancet Infect Dis. (. 2024) 24:285–96. doi: 10.1016/s1473-3099(23)00501-7, PMID: 38012890 PMC11876094

[43] Zhu B Dockrell HM Ottenhoff THM Evans TG Zhang Y. Tuberculosis vaccines: Opportunities and challenges. Respirology. (2018) 23:359–68. doi: 10.1111/resp.13245, PMID: 29341430

[44] Gong W Liang Y Wu X. The current status, challenges, and future developments of new tuberculosis vaccines. Hum Vaccin Immunother. (2018) 14:1697–716. doi: 10.1080/21645515.2018.1458806, PMID: 29601253 PMC6067889

[45] Talwar GP Gupta JC Mustafa AS Kar HK Katoch K Parida SK. Development of a potent invigorator of immune responses endowed with both preventive and therapeutic properties. Biologics. (2017) 11:55–63. Available online at: https://www.dovepress.com/development-of-a-potent-invigorator-of-immune-responses-endowed-with-b-peer-reviewed-fulltext-article-BTT (Accessed March 13, 2025)., PMID: 28496303 10.2147/BTT.S128308 PMC5422320

[46] Sharma A Saqib M Sheikh JA Ehtesham NZ Bhaskar S Chaudhuri TK. Mycobacterium indicus pranii protein MIP_05962 induces Th1 cell mediated immune response in mice. Int J Med Microbiol. (2018) 308:1000–8. doi: 10.1016/j.ijmm.2018.08.008, PMID: 30190103

[47] Saqib M Khatri R Singh B Gupta A Kumar A Bhaskar S. Mycobacterium indicus pranii as a booster vaccine enhances BCG induced immunity and confers higher protection in animal models of tuberculosis. Tuberculosis. (2016) 101:164–73. doi: 10.1016/j.tube.2016.10.002, PMID: 27865389

[48] Singh B Saqib M Gupta A Kumar P Bhaskar S. Autophagy induction by Mycobacterium indicus pranii promotes Mycobacterium tuberculosis clearance from RAW 264.7 macrophages. PloS One. (2017) 12:e0189606. doi: 10.1371/journal.pone.0189606, PMID: 29236768 PMC5728553

[49] Kumar P Das G Bhaskar S. Mycobacterium indicus pranii therapy induces tumor regression in MyD88- and TLR2-dependent manner. BMC Res Notes. (2019) 12:1–5. doi: 10.1186/s13104-019-4679-0, PMID: 31590685 PMC6781299

[50] Saqib M Khatri R Singh B Gupta A Bhaskar S. Cell wall fraction of Mycobacterium indicus pranii shows potential Th1 adjuvant activity. Int Immunopharmacol. (2019) 70:408–16. doi: 10.1016/j.intimp.2019.02.049, PMID: 30856391

[51] Kumar P John V Marathe S Das G Bhaskar S. Mycobacterium indicus pranii induces dendritic cell activation, survival, and Th1/Th17 polarization potential in a TLR-dependent manner. J Leukoc Biol. (2015) 97:511–20. doi: 10.1189/jlb.1A0714-361R, PMID: 25593326 PMC5477886

[52] Katoch K Singh P Adhikari T Benara SK Singh HB Chauhan DS. Potential of Mw as a prophylactic vaccine against pulmonary tuberculosis. Vaccine. (2008) 26:1228–34. doi: 10.1016/j.vaccine.2007.12.025, PMID: 18243430

[53] Dlugovitzky D Fiorenza G Farroni M Bogue C Stanford C Stanford J. Immunological consequences of three doses of heat-killed Mycobacterium vaccae in the immunotherapy of tuberculosis. Respir Med. (2006) 100:1079–87. doi: 10.1016/j.rmed.2005.09.026, PMID: 16278080

[54] Cardona PJ. RUTI: A new chance to shorten the treatment of latent tuberculosis infection. Tuberculosis. (2006) 86:273–89. doi: 10.1016/j.tube.2006.01.024, PMID: 16545981

[55] Triccas JA Counoupas C Damoulari C Cardona PJ Fabregat AM P-j C. Efficacy of RUTI^®^ immunotherapy against active tuberculosis in a mouse model challenges the Koch phenomenon. Front Tuberculosis. (2023) 1:1240684. doi: 10.3389/ftubr.2023.1240684 PMC13494941 42638627

[56] Stucki D Brites D Jeljeli L Coscolla M Liu Q Trauner A. Mycobacterium tuberculosis lineage 4 comprises globally distributed and geographically restricted sublineages. Nat Genet. (2016) 48:1535–43:12. Available online at: https://www.nature.com/articles/ng.3704 (Accessed March 10, 2025)., PMID: 27798628 10.1038/ng.3704 PMC5238942

[57] Arbues A Aguilo JI Gonzalo-Asensio J Marinova D Uranga S Puentes E. Construction, characterization and preclinical evaluation of MTBVAC, the first live-attenuated M. tuberculosis-based Vaccine to enter Clin trials. Vaccine. (2013) 31:4867–73. doi: 10.1016/j.vaccine.2013.07.051, PMID: 23965219

[58] White AD Sibley L Sarfas C Morrison A Gullick J Clark S. MTBVAC vaccination protects rhesus macaques against aerosol challenge with M. tuberculosis and induces immune signatures analogous to those observed in clinical studies. NPJ Vaccines. (2021) 6. doi: 10.1038/s41541-020-00262-8, PMID: 33397991 PMC7782851

[59] Aguilo N Uranga S Marinova D Monzon M Badiola J Martin C. MTBVAC vaccine is safe, immunogenic and confers protective efficacy against Mycobacterium tuberculosis in newborn mice. Tuberculosis. (2016) 96:71–4. doi: 10.1016/j.tube.2015.10.010, PMID: 26786657 PMC4727503

[60] Clark S Lanni F Marinova D Rayner E Martin C Williams A. Revaccination of Guinea Pigs With the Live Attenuated Mycobacterium tuberculosis Vaccine MTBVAC Improves BCG’s Protection Against Tuberculosis. J Infect Dis J Infect Dis ®. (2017) 525:525–58. Available online at: https://academic.oup.com/jid/article/216/5/525/2949726 (Accessed February 26, 2025)., PMID: 28329234 10.1093/infdis/jix030

[61] Pollard AJ Finn A Curtis N. Non-specific effects of vaccines: plausible and potentially important, but implications uncertain. Arch Dis Child. (2017) 102:1077–81. Available online at: https://adc.bmj.com/content/102/11/1077 (Accessed March 14, 2025)., PMID: 28501809 10.1136/archdischild-2015-310282

[62] Higgins JPT Soares-Weiser K López-López JA Kakourou A Chaplin K Christensen H. Association of BCG, DTP, and measles containing vaccines with childhood mortality: systematic review. BMJ. (2016) 355:5170. Available online at: https://www.bmj.com/content/355/bmj.i5170 (Accessed March 14, 2025)., PMID: 27737834 10.1136/bmj.i5170 PMC5063034

[63] Lancione S Alvarez JV Alsdurf H Pai M Zwerling AA. Tracking changes in national BCG vaccination policies and practices using the BCG World Atlas. BMJ Glob Health. (2022) 7:7462. Available online at: https://gh.bmj.com/content/7/1/e007462 (Accessed March 14, 2025)., PMID: 35039309 10.1136/bmjgh-2021-007462 PMC8764994

[64] Nieuwenhuizen NE Kaufmann SHE. Next-generation vaccines based on Bacille Calmette-Guérin. Front Immunol. (2018) 9:325109. Available online at: www.frontiersin.org (Accessed March 2, 2025)., PMID: 29459859 10.3389/fimmu.2018.00121 PMC5807593

[65] Wu Y Cai M Ma J Teng X Tian M Bassuoney EBMB. Heterologous boost following mycobacterium bovis BCG reduces the late persistent, rather than the early stage of intranasal tuberculosis challenge infection. Front Immunol. (2018) 9:414489. doi: 10.3389/fimmu.2018.02439, PMID: 30425711 PMC6218689

[66] Ma J Teng X Wang X Fan X Wu Y Tian M. A multistage subunit vaccine effectively protects mice against primary progressive tuberculosis, latency and reactivation. EBioMedicine. (2017) 22:143–54. Available online at: https://www.thelancet.com/action/showFullText?pii=S2352396417302682 (Accessed March 14, 2025)., PMID: 28711483 10.1016/j.ebiom.2017.07.005 PMC5552207

[67] Lei Q Fu H Yao Z Zhou Z Wang Y Lin X. Early introduction of IL-10 weakens BCG revaccination’s protection by suppressing CD4+Th1 cell responses. J Transl Med. (2024) 22:1–14. doi: 10.1186/s12967-024-05683-w, PMID: 39633471 PMC11616166

[68] Nemes E Hesseling AC Tameris M Mauff K Downing K Mulenga H. Safety and immunogenicity of newborn MVA85A vaccination and selective, delayed bacille calmette-guerin for infants of human immunodeficiency virus-infected mothers: A phase 2 randomized, controlled trial. Clin Infect Dis. (. 2018) 66:554–63. doi: 10.1093/cid/cix834, PMID: 29028973 PMC5849090

[69] Lawrence A. Bacillus calmette-guérin (BCG) revaccination and protection against tuberculosis: A systematic review. San Francisco, California: Cureus (2024). 10.7759/cureus.56643 PMC11032142 38646352

[70] Ohara N Yamada T. Recombinant BCG vaccines. Vaccine. (2001) 19:4089–98. doi: 10.1016/s0264-410x(01)00155-4, PMID: 11457532

[71] Nieuwenhuizen NE Kulkarni PS Shaligram U Cotton MF Rentsch CA Eisele B. The recombinant bacille Calmette-Guérin vaccine VPM1002: Ready for clinical efficacy testing. Front Immunol. (2017) 8:292743. Available online at: www.frontiersin.org (Accessed March 7, 2025)., PMID: 28974949 10.3389/fimmu.2017.01147 PMC5610719

[72] Gengenbacher M Nieuwenhuizen N Vogelzang A Liu H Kaiser P Schuerer S. Deletion of nuoG from the vaccine candidate Mycobacterium bovis BCG ΔureC:: Hly improves protection against tuberculosis. mBio. (2016) 7:e00679-16. doi: 10.1128/mbio.00679-16, PMID: 27222470 PMC4895111

[73] Griffiths KL Ahmed M Das S Gopal R Horne W Connell TD. Targeting dendritic cells to accelerate T-cell activation overcomes a bottleneck in tuberculosis vaccine efficacy. Nat Commun. (2016) 7:1. Available online at: https://www.nature.com/articles/ncomms13894 (Accessed March 14, 2025)., PMID: 28004802 10.1038/ncomms13894 PMC5192216

[74] Grode L Ganoza CA Brohm C Weiner J Eisele B Kaufmann SHE. Safety and immunogenicity of the recombinant BCG vaccine VPM1002 in a phase 1 open-label randomized clinical trial. Vaccine. (2013) 31:1340–8. doi: 10.1016/j.vaccine.2012.12.053, PMID: 23290835

[75] Ouaked N Demoitié MA Godfroid F Mortier MC Vanloubbeeck Y Temmerman ST. Non-clinical evaluation of local and systemic immunity induced by different vaccination strategies of the candidate tuberculosis vaccine M72/AS01. Tuberculosis. (2023) 143:102425. doi: 10.1016/j.tube.2023.102425, PMID: 38180028

[76] Williams A Orme IM. Animal models of tuberculosis: an overview. Microbiol Spectr. (2016) 4. doi: 10.1128/microbiolspec.tbtb2-0004-2015, PMID: 27726810

[77] Ji Z Jian M Chen T Luo L Li L Dai X. Immunogenicity and safety of the M72/AS01E candidate vaccine against tuberculosis: A meta-analysis. Front Immunol. (2019) 10:2089. Available online at: https://pmc.ncbi.nlm.nih.gov/articles/PMC6735267/., PMID: 31552037 10.3389/fimmu.2019.02089 PMC6735267

[78] Rodo MJ Rozot V Nemes E Dintwe O Hatherill M Little F. comparison of antigen-specific T cell responses induced by six novel tuberculosis vaccine candidates. PloS Pathog. (2019) 15:e1007643. doi: 10.1371/journal.ppat.1007643, PMID: 30830940 PMC6417742

[79] Agger EM. Novel adjuvant formulations for delivery of anti-tuberculosis vaccine candidates. Adv Drug Delivery Rev. (2016) 102:73–82. doi: 10.1016/j.addr.2015.11.012, PMID: 26596558 PMC4870161

[80] Tkachuk AP Gushchin VA Potapov VD Demidenko AV Lunin VG Gintsburg AL. Multi-subunit BCG booster vaccine GamTBvac: Assessment of immunogenicity and protective efficacy in murine and Guinea pig TB models. PloS One. (. 2017) 12:e0176784. doi: 10.1371/journal.pone.0176784, PMID: 28453555 PMC5409163

[81] Coler RN Bertholet S Moutaftsi M Guderian JA Windish HP Baldwin SL. Development and characterization of synthetic glucopyranosyl lipid adjuvant system as a vaccine adjuvant. PloS One. (2011) 6:e16333. doi: 10.1371/journal.pone.0016333, PMID: 21298114 PMC3027669

[82] Baldwin SL Reese VA Huang PWD Beebe EA Podell BK Reed SG. Protection and Long-Lived Immunity Induced by the ID93/GLA-SE Vaccine Candidate against a Clinical Mycobacterium tuberculosis Isolate. . Clin Vaccine Immunol. (2016) 23:137–47. doi: 10.1128/CVI.00458-15, PMID: 26656121 PMC4744918

[83] Baldwin SL Reese VA Larsen SE Beebe E Guderian J Orr MT. Prophylactic efficacy against Mycobacterium tuberculosis using ID93 and lipid-based adjuvant formulations in the mouse model. PloS One. (2021) 16:e0247990. doi: 10.1371/journal.pone.0247990, PMID: 33705411 PMC7951850

[84] GLOBAL TUBERCULOSIS REPORT 2020 (2020). Available online at: http://apps.who.int/bookorders (Accessed Mar 15, 2025).

[85] Guo X Lu J Li J Du W Shen X Su C. The Subunit AEC/BC02 Vaccine Combined with Antibiotics Provides Protection in Mycobacterium tuberculosis-Infected Guinea Pigs. Vaccines (Basel). (2022) 10:2164. Available online at: https://pmc.ncbi.nlm.nih.gov/articles/PMC9781032/., PMID: 36560574 10.3390/vaccines10122164 PMC9781032

[86] Lu J Guo X Wang C Du W Shen X Su C. Therapeutic Effect of Subunit Vaccine AEC/BC02 on Mycobacterium tuberculosis Post-Chemotherapy Relapse Using a Latent Infection Murine Model. Vaccines. (2022) 10:825. Available online at: https://www.mdpi.com/2076-393X/10/5/825/htm (Accessed March 8, 2025)., PMID: 35632581 10.3390/vaccines10050825 PMC9145927

[87] McCann N O’Connor D Lambe T Pollard AJ. Viral vector vaccines. Curr Opin Immunol. (2022) 77:102210. doi: 10.1016/j.coi.2022.102210, PMID: 35643023 PMC9612401

[88] Williams A Goonetilleke NP McShane H Clark SO Hatch G Gilbert SC. Boosting with poxviruses enhances Mycobacterium bovis BCG efficacy against tuberculosis in Guinea pigs. Infect Immun. (2005) 73:3814–6. doi: 10.1128/iai.73.6.3814-3816.2005, PMID: 15908420 PMC1111825

[89] Verreck FAW Vervenne RAW Kondova I van Kralingen KW Remarque EJ Braskamp G. MVA.85A Boosting of BCG and an Attenuated, phoP Deficient M. tuberculosis Vaccine Both Show Protective Efficacy Against Tuberculosis in Rhesus Macaques. PloS One. (2009) 4:e5264. doi: 10.1371/journal.pone.0005264, PMID: 19367339 PMC2666807

[90] Vordermeier HM Villarreal-Ramos B Cockle PJ McAulay M Rhodes SG Thacker T. Viral booster vaccines improve Mycobacterium bovis BCG-induced protection against bovine tuberculosis. Infect Immun. (2009) 77:3364–73. doi: 10.1128/iai.00287-09, PMID: 19487476 PMC2715681

[91] McShane H Brookes R Gilbert SC Hill AVS. Enhanced immunogenicity of CD4+ T-cell responses and protective efficacy of a DNA-modified vaccinia virus ankara prime-boost vaccination regimen for murine tuberculosis. Infect Immun. (2001) 69:681–6. doi: 10.1128/iai.69.2.681-686.2001, PMID: 11159955 PMC97939

[92] Hawkridge T Scriba TJ Gelderbloem S Smit E Tameris M Moyo S. Safety and immunogenicity of a new tuberculosis vaccine, MVA85A, in healthy adults in South Africa. J Infect Dis. (2008) 198:544–52. doi: 10.1086/590185, PMID: 18582195 PMC2822902

[93] Scriba TJ Tameris M Mansoor N Smit E van der Merwe L Mauff K. Dose-finding study of the novel tuberculosis vaccine, MVA85A, in healthy BCG-vaccinated infants. J Infect Dis. (2011) 203:1832–43. doi: 10.1093/infdis/jir195, PMID: 21606542

[94] Voysey M Costa Clemens SA Madhi SA Weckx LY Folegatti PM Aley PK. Single-dose administration and the influence of the timing of the booster dose on immunogenicity and efficacy of ChAdOx1 nCoV-19 (AZD1222) vaccine: a pooled analysis of four randomised trials. Lancet. (2021) 397:881–91. Available online at: https://www.thelancet.com/action/showFullText?pii=S0140673621004323 (Accessed March 13, 2025)., PMID: 33617777 10.1016/S0140-6736(21)00432-3 PMC7894131

[95] Stylianou E Griffiths KL Poyntz HC Harrington-Kandt R Dicks MD Stockdale L. Improvement of BCG protective efficacy with a novel chimpanzee adenovirus and a modified vaccinia Ankara virus both expressing Ag85A. Vaccine. (2015) 33:6800–8. doi: 10.1016/j.vaccine.2015.10.017, PMID: 26478198 PMC4678294

[96] Lipsitch M Dean NE. Understanding COVID-19 vaccine efficacy. Science. (1979) 370:763–5. doi: 10.1126/science.abe5938?download=true 33087460

[97] Zimmermann P Curtis N. Factors that influence the immune response to vaccination. Clin Microbiol Rev. (2019) 32. doi: 10.1128/cmr.00084-18?download=true PMC6431125 30867162

[98] Falahi S Kenarkoohi A. Host factors and vaccine efficacy: Implications for COVID-19 vaccines. J Med Virol. (2022) 94:1330–5. doi: 10.1002/jmv.27485, PMID: 34845730 PMC9015327

[99] Dhakal S Klein SL. Host factors impact vaccine efficacy: implications for seasonal and universal influenza vaccine programs. J Virol. (2019) 93. doi: 10.1128/jvi.00797-19, PMID: 31391269 PMC6803252

[100] Tsang TK Wang C Tsang NNY Fang VJ Perera RAPM Malik Peiris JS. Impact of host genetic polymorphisms on response to inactivated influenza vaccine in children. NPJ Vaccines. (2023) 8:1. Available online at: https://www.nature.com/articles/s41541-023-00621-1 (Accessed March 17, 2025)., PMID: 36804941 10.1038/s41541-023-00621-1 PMC9940051

[101] Lai R Gong DN Williams T Ogunsola AF Cavallo K Arlehamn CSL. Host genetic background is a barrier to broadly effective vaccine-mediated protection against tuberculosis. J Clin Invest. (2023) 133:e167762. doi: 10.1172/jci167762, PMID: 37200108 PMC10313364

[102] De Albuquerque AC Rocha LQ De Morais Batista AH Teixeira AB Dos Santos DB Nogueira NAP. Association of polymorphism +874 A/T of interferon-γ and susceptibility to the development of tuberculosis: Meta-analysis. Eur J Clin Microbiol Infect Diseases. (2012) 31:2887–95. doi: 10.1007/s10096-012-1660-4, PMID: 22684265

[103] Sallakci N Coskun M Berber Z Gürkan F Kocamaz H Uysal G. Interferon-γ gene+874T–A polymorphism is associated with tuberculosis and gamma interferon response. Tuberculosis. (2007) 87:225–30. doi: 10.1016/j.tube.2006.10.002, PMID: 17276141

[104] Anuradha B Rakh SS Ishaq M Murthy KJR Valluri VL. Interferon-gamma Low producer genotype +874 overrepresented in Bacillus Calmette-Guerin nonresponding children. Pediatr Infect Dis J. (2008) 27:325–9. doi: 10.1097/INF.0b013e31816099e6, PMID: 18379373

[105] Leung AS Tran V Wu Z Yu X Alexander DC Gao GF. Novel genome polymorphisms in BCG vaccine strains and impact on efficacy. BMC Genomics. (2008) 9:1–12. doi: 10.1186/1471-2164-9-413, PMID: 18793412 PMC2553098

[106] Jabot-Hanin F Cobat A Feinberg J Orlova M Niay J Deswarte C. An eQTL variant of ZXDC is associated with IFN-γ production following Mycobacterium tuberculosis antigen-specific stimulation. Sci Rep. (2017) 7:1. Available online at: https://www.nature.com/articles/s41598-017-13017-8 (Accessed March 17, 2025)., PMID: 28993696 10.1038/s41598-017-13017-8 PMC5634485

[107] Kimmey JM Huynh JP Weiss LA Park S Kambal A Debnath J. Unique role for ATG5 in neutrophil-mediated immunopathology during M. tuberculosis infection. Nat. (2015) 528:7583. Available online at: https://www.nature.com/articles/nature16451 (Accessed March 12, 2025)., PMID: 26649827 10.1038/nature16451 PMC4842313

[108] Philips JA Porto MC Wang H Rubin EJ Perrimon N. ESCRT factors restrict mycobacterial growth. Proc Natl Acad Sci U S A. (2008) 105:3070–5. doi: 10.1073/pnas.0707206105, PMID: 18287038 PMC2268586

[109] Feng CG Scanga CA Collazo-Custodio CM Cheever AW Hieny S Caspar P. Mice lacking myeloid differentiation factor 88 display profound defects in host resistance and immune responses to mycobacterium avium infection not exhibited by toll-like receptor 2 (TLR2)- and TLR4-deficient animals. J Immunol. (2003) 171:4758–64. doi: 10.4049/jimmunol.171.9.4758, PMID: 14568952

[110] Mayer-Barber KD Andrade BB Oland SD Amaral EP Barber DL Gonzales J. Host-directed therapy of tuberculosis based on interleukin-1 and type I interferon crosstalk. Nature. (2014) 511:7507. Available online at: https://www.nature.com/articles/nature13489 (Accessed March 12, 2025)., PMID: 24990750 10.1038/nature13489 PMC4809146

[111] Shah JA Vary JC Chau TT Bang ND Yen NT Farrar JJ. Human TOLLIP regulates TLR2 and TLR4 signaling and its polymorphisms are associated with susceptibility to tuberculosis. J Immunol. (2012) 189:1737–46. doi: 10.4049/jimmunol.1103541, PMID: 22778396 PMC3428135

[112] Graustein AD Misch EA Musvosvi M Shey M Shah JA Seshadri C. Toll-like receptor chaperone HSP90B1 and the immune response to Mycobacteria. PloS One. (2018) 13:e0208940. doi: 10.1371/journal.pone.0208940, PMID: 30550567 PMC6294361

[113] Shah JA Musvosvi M Shey M Horne DJ Wells RD Peterson GJ. A functional toll-interacting protein variant is associated with bacillus calmette-guérin-specific immune responses and tuberculosis. Am J Respir Crit Care Med. (2017) 196:502–11. Available online at: http://grch37.ensembl.org/index (Accessed March 9, 2025)., PMID: 28463648 10.1164/rccm.201611-2346OC PMC5564674

[114] Aguilo N Gonzalo-Asensio J Alvarez-Arguedas S Marinova D Gomez AB Uranga S. Reactogenicity to major tuberculosis antigens absent in BCG is linked to improved protection against Mycobacterium tuberculosis. Nat Commun. (2017) 8:16085. Available online at: https://pmc.ncbi.nlm.nih.gov/articles/PMC5519979/., PMID: 28706226 10.1038/ncomms16085 PMC5519979

[115] Lindestam Arlehamn CS McKinney DM Carpenter C Paul S Rozot V Makgotlho E. A quantitative analysis of complexity of human pathogen-specific CD4 T cell responses in healthy M. tuberculosis infected South Africans. PloS Pathog. (2016) 12:1005760. doi: 10.1371/journal.ppat.1005760, PMID: 27409590 PMC4943605

[116] Randhawa AK Shey MS Keyser A Peixoto B Wells RD de Kock M. Association of human TLR1 and TLR6 deficiency with altered immune responses to BCG vaccination in South African infants. PloS Pathog. (2011) 7:e1002174. doi: 10.1371/journal.ppat.1002174, PMID: 21852947 PMC3154845

[117] Pöyhönen L Kröger L Huhtala H Mäkinen J Nuolivirta K Mertsola J. Association of MBL2, TLR1, TLR2 and TLR6 polymorphisms with production of IFN-γ and IL-12 in BCG osteitis survivors R1. Pediatr Infect Dis J. (2017) 36:135–9. doi: 10.1097/inf.0000000000001375, PMID: 27755461

[118] Abdallah AM Hill-Cawthorne GA Otto TD Coll F Guerra-Assunção JA Gao G. Genomic expression catalogue of a global collection of BCG vaccine strains show evidence for highly diverged metabolic and cell-wall adaptations. Sci Rep. (2015) 5:1. Available online at: https://www.nature.com/articles/srep15443 (Accessed March 13, 2025)., PMID: 26487098 10.1038/srep15443 PMC4614345

[119] Price DN Kusewitt DF Lino CA McBride AA Muttil P. Oral Tolerance to Environmental Mycobacteria Interferes with Intradermal, but Not Pulmonary, Immunization against Tuberculosis. PloS Pathog. (2016) 12:e1005614. doi: 10.1371/journal.ppat.1005614, PMID: 27153120 PMC4859477

[120] Boyd SD Jackson KJL. Predicting vaccine responsiveness. Cell Host Microbe. (2015) 17:301–7. Available online at: https://www.cell.com/action/showFullText?pii=S1931312815000724 (Accessed March 13, 2025). 10.1016/j.chom.2015.02.015 25766292

[121] Brodin P Jojic V Gao T Bhattacharya S Angel CJL Furman D. Variation in the human immune system is largely driven by non-herita ble influences. Cell. (2015) 160:37–47. Available online at: https://www.cell.com/action/showFullText?pii=S0092867414015906 (Accessed March 13, 2025)., PMID: 25594173 10.1016/j.cell.2014.12.020 PMC4302727

[122] Jabot-Hanin F Cobat A Feinberg J Grange G Remus N Poirier C. Major loci on chromosomes 8q and 3q control interferon γ Production triggered by bacillus calmette-guerin and 6-kDa early secretory antigen target, respectively, in various populations. J Infect Dis. (2016) 213:1173–9. doi: 10.1093/infdis/jiv757, PMID: 26690346 PMC4779307

[123] Smith CM Proulx MK Olive AJ Laddy D Mishra BB Moss C. Tuberculosis susceptibility and vaccine protection are independently controlled by host genotype. MBio. (2016) 7:e01516-16. doi: 10.1128/mbio.01516-16, PMID: 27651361 PMC5030360

[124] Churchyard GJ Houben RMGJ Fielding K Fiore-Gartland AL Esmail H Grant AD. Implications of subclinical tuberculosis for vaccine trial design and global effect. Lancet Microbe. (2024) 5:100895. Available online at: https://www.thelancet.com/action/showFullText?pii=S2666524724001277 (Accessed March 9, 2025)., PMID: 38964359 10.1016/S2666-5247(24)00127-7 PMC11464400

[125] Prabowo SA Painter H Zelmer A Smith SG Seifert K Amat M. Vaccination Enhances Inhibition of Mycobacterial Growth ex vivo and Induces a Shift of Monocyte Phenotype in Mice. Front Immunol. (2019) 10. doi: 10.3389/fimmu.2019.00894, PMID: 31114572 PMC6503078

