# Correction: BCG and beyond: unlocking new frontiers in TB vaccine development

**DOI:** 10.3389/fimmu.2025.1677868

**Published:** 2025-08-25

**Authors:** Aishwarya Shaji, Akanksha Verma, Ashima Bhaskar, Ved Prakash Dwivedi

**Affiliations:** Immunobiology Group, International Centre for Genetic Engineering and Biotechnology, New Delhi, India

**Keywords:** tuberculosis, BCG, trained immunity, vaccine, host genetics

In the **Abstract** irrelevant text was inserted erroneously during production.

The **Abstract** has been corrected to read:

“With over 10 million new cases and 1.6 million deaths annually, tuberculosis (TB) continues to be a significant worldwide health-burden. To assist in curbing the spread of TB, the century-old BCG, which is a live-attenuated vaccine, is now the only licensed TB vaccine used in humans. However, BCG’s limited efficacy and poor antigenicity in adults have evoked the need to design new vaccines against TB. The limited parameter is the availability of potent antigens; as a consequence, it is imperative to study the Mycobacterium tuberculosis (Mtb)-specific antigens that can provide a stronger immune response if included in vaccine candidates. Through this review, we aim to concentrate on the progress of current vaccine-candidates undergoing preclinical and clinical-studies. Moreover, it is not the pathogen but the genetics of the host that plays an essential role in fine tuning the immune-response and susceptibility to TB. Over the past 50 years, a systematic approach to treating TB patients has overlooked factors like pharmacokinetics, immune-response, and treatment duration. Henceforth, this review highlights the precision medicine-guided approach considering genetic makeup and host immunity that could influence clinical management choices. The consolidated review will shed light on advancements in vaccine-candidates, which can be harnessed in prophylactic development against TB.’’

The original version of this article has been updated.

